# Entwicklung des Einsamkeitsempfindens im Alter in Zeiten von COVID-19: Gruppenvergleiche älterer Menschen vor und in der ersten Welle der Pandemie

**DOI:** 10.1007/s11614-021-00461-0

**Published:** 2021-12-21

**Authors:** Lukas Richter, Theresa Heidinger

**Affiliations:** 1 Department Soziales, Fachhochschule St. Pölten, Matthias Corvinus-Straße 15, 3100 St. Pölten, Österreich; 2 Institut für Soziologie und empirische Sozialforschung, Wirtschaftsuniversität Wien, Wien, Österreich; 3 Kompetenzzentrum Gerontologie und Gesundheitsforschung, Karl Landsteiner Privatuniversität, Krems, Österreich

**Keywords:** Covid-19, Ältere Menschen, Einsamkeit, Entwicklung der Einsamkeit, Sozioökonomische Faktoren, Gruppenvergleiche, Covid-19, Older people, Loneliness, Development of loneliness, Socio-economic factors, Group comparisons

## Abstract

Der Beitrag betrachtet die Einsamkeitsentwicklung vor und in den ersten Monaten der Covid-19-Pandemie vor dem Hintergrund sozioökonomischer Lagen älterer Menschen. Hierfür werden Varianzanalysen mit Daten aus Niederösterreich aus zwei Surveys (vor und während der Covid-19-Pandemie) durchgeführt. Die Analysen zeigen, dass es sich bei älteren Menschen nicht um eine homogene Gruppe handelt, das Einsamkeitsniveau also bereits vor der Pandemie divergent ausgeprägt war und sich diese bestehenden Unterschiede während der Pandemie mit geringer Variation weitgehend auf einem etwas höheren Niveau erhalten haben.

## Abstract (en)

The article investigates the development of loneliness before and in the first months of the Covid-19 pandemic against the background of the socio-economic situation of older people. For this purpose, variance analyses are carried out with data from Lower Austria utilizing two surveys (pre-pandemic, peri-pandemic). The analyses show that older people are not a homogeneous group, i.e. the level of loneliness was already divergent before the pandemic and these existing differences were largely maintained at a somewhat higher level during the pandemic with little variation.

## Einleitung und Forschungsziel

Bereits mit Beginn der Covid-19 Pandemie wurde vor einer Zunahme der Einsamkeit insbesondere aufgrund der Distanzierungsmaßnahmen seitens der Wissenschaft gewarnt (Armitage und Nellums 2020; Banerjee und Rai 2020; Brooke und Jackson 2020; Chatterjee et al. 2020). Relevanz erhält das Thema, da Einsamkeit – als subjektiv empfundene Diskrepanz zwischen der erwarteten und tatsächlichen Qualität und Quantität von sozialen Beziehungen oder als Ausdruck von negativen Gefühlen fehlender Beziehungen definiert (de Jong Gierveld und van Tilburg 2006) – mit einer reduzierten Lebensqualität (Musich et al. 2015) sowie erhöhten Morbiditätsrisiken (Courtin und Knapp 2017) in Verbindung steht. Unter anderem ist Einsamkeit mit psychischen Krankheiten wie Depression oder Angst (Beutel et al. 2017) und physischen Krankheiten wie Herz-Kreislauf-Erkrankungen (Valtorta et al. 2016) assoziiert und gilt – ähnlich wie Adipositas und Nikotinmissbrauch (Holt-Lunstad et al. 2015) als Mortalitätsrisiko (Lara et al. 2020; Steptoe et al. 2013).

Mittlerweile hat sich der vermutete und quer durch die Bevölkerung ziehende Anstieg der Einsamkeit im Zuge der Pandemie und der einhergehenden Distanzierungsmaßnahmen in einer wachsenden Zahl an internationalen Studien bestätigt (u. a. Bu et al. 2020; Groarke et al. 2020; Varga et al. 2021). Obgleich eine höhere Einsamkeitsprävalenz bei jüngeren als bei ältere Menschen während der Pandemie verzeichnet werden kann, ist eine Zunahmen bei älteren Menschen ebenso zu bescheinigen (Entringer et al. 2020; Heidinger und Richter 2020; Luchetti et al. 2020; Carson et al. 2020; Krendl und Perry 2021; Creese et al. 2020). Allerdings wurden in den genannten Studien ältere Personen rein aufgrund des Merkmals „Alter“ gruppiert und analysiert, während soziodemografisch differenziertere Analysen fragmentiert und weiterhin selten zu finden sind (Lebrasseur et al. 2021; van Tilburg et al. 2020). Dies ist aus sozio-gerontologischer Sicht kritisch zu betrachten, da es sich bei älteren Menschen um eine heterogene Gruppe handelt, welche sich aufgrund unterschiedlicher Fähigkeiten und Einschränkungen, Biografien und Lebensstile (Wahl und Heyl 2015), wie sozioökonomischer Ressourcen und letztlich Handlungsmöglichkeiten, ausdifferenziert. Vor diesem Hintergrund zeigen Studien (Cohen-Mansfield et al. 2016; Luhmann und Hawkley 2016; Victor et al. 2005), dass innerhalb der Gruppe der älteren Menschen Frauen, Personen mit niedrigerem Einkommen und Bildungsstatus sowie Personen mit einem schlechten Gesundheitszustand ein höheres Risiko für Einsamkeit haben. Ebenso sind ältere Menschen mit geringer sozialer Eingebundenheit und einer geringeren Zahl an nahestehenden Personen oder Alleinstehende und alleinlebende Personen einsamer. Wong et al. (2020) kommen mittels multivariater Analyse zum Schluss, dass auch während der Pandemie ältere alleinlebende (auch Stolz et al. 2020) bzw. multimorbide Personen sowie ältere Frauen ein signifikant höheres Einsamkeitsniveau haben – ebenso Whatley et al. (2020), welche neben Geschlecht und Gesundheit auf einen signifikant negativen Zusammenhang zwischen Einkommen und Einsamkeit während der Pandemie verweisen (auch Stickley et al. 2021) –, ohne jedoch die Entwicklung der gruppenspezifischen Unterschiede zu betrachten, also wie sich das Niveau der Einsamkeit innerhalb beispielsweise der Geschlechter entwickelte und ob sich damit das Ausmaß des Unterschieds zwischen Männern und Frauen vor und während der Pandemie verändert hat. Des Weiteren ist anzumerken, dass sich aktuell der Stand der Forschung zum Thema Einsamkeit und Pandemie rasch entwickelt, weiterhin aber ein explorativer Charakter vorfindlich ist und vorrangig Ad-hoc-Annahmen zu attestieren sind (Dahlberg 2021). Vermutet wird zum Beispiel, dass Differenzen in der Einsamkeitsentwicklung während der Pandemie zwischen den Altersgruppen auf geringere Änderungen im Tagesablauf im Vergleich zu erwerbstätigen, jüngeren Erwachsenen oder auf eine höhere Resilienz, bessere Regulierung von Emotionen und bessere Bewältigungsstrategien bei älteren Menschen zurückzuführen sind (Lebrasseur et al. 2021). Nicht überraschend werden die sozialen Distanzierungsmaßnahmen und damit die Abnahme sozialer Kontaktmöglichkeiten für die Zunahme der Einsamkeit verantwortlich gehalten (u. a. Armitage und Nellums 2020), wobei materielle und immaterielle Ressourcen als potenzielle Puffer dienen dürften, aber gleichzeitig der Rückgang der sozialen Kontakte bei Gruppen mit höherem Ausgangsniveau zu einem überproportionalen Anstieg des Gefühls der Einsamkeit führen könnte (Dahlberg 2021). Erste Studien konnten zeigen, dass alleinlebende ältere Menschen (Emerson 2020) und ältere Frauen (Wickens et al. 2021) signifikant öfter von gehäuften Einsamkeitsgefühlen (Quantität) berichten, was sich in einem höheren Einsamkeitsniveau niederschlagen müsste (Qualität).

Zusammenfassend ist eine Zunahme der Einsamkeit während der Pandemie bei älteren Menschen sehr wahrscheinlich, wobei sich Differenzen zwischen den sozioökonomischen Gruppen teilweise fortgeschrieben haben dürften oder es gar, wie aus letztgenannten Studien abgeleitet werden kann, teils zu einer Ausweitung der Differenzen im Einsamkeitsniveau gekommen sein könnte. Die Ergebnisse sind bis dato jedoch fragmentiert, teils Nebenprodukte anderer Fragestellungen bzw. wurden, nach Erkenntnisstand der AutorInnen, Faktoren, wie die soziale Unterstützung, noch nicht in Publikationen berücksichtigt. Ziel der Arbeit ist es daher, mittels Varianzanalysen die Einsamkeitsentwicklung *vor* und *in den ersten Monaten der Pandemie* vor dem Hintergrund sozioökonomischer Lagen älterer Menschen zu analysieren. Hiermit soll geprüft werden, ob ältere Menschen allgemein einsamer wurden oder ob bestimmte Gruppen divergent betroffen waren bzw. ob es im Vergleich der Zeitpunkte zu Ausweitungen, Konvergenzen oder Fortschreibungen der bestehenden Differenzen im Einsamkeitsniveau kam.

## Studiendesign

Zur Bildung der longitudinalen Studienstichprobe sind zwei unabhängig gezogene, repräsentative Datensätze kombiniert worden: a) das Gesundheitsbarometer Alter NÖ (t_0_) als präpandemische Umfrage, die zwischen April und Juli 2019 durchgeführt wurde (*n* = 2042), und b) die Covid-19 Alter NÖ Befragung (t_1_) als peri-pandemische Umfrage, mit einem Erhebungszeitraum zwischen April und Mai 2020 (*n* = 521). Bei beiden Erhebungen handelt es sich um Telefonbefragungen der älteren niederösterreichischen Bevölkerung (60+) in Privathaushalten. Das Sampling erfolgte jeweils nach Gemeindegrößen über eine vorab geschichtete Zufallsauswahl mit Altersscreening. Um Vergleichbarkeit zwischen den Surveys herzustellen, wurde ein Teil der Items harmonisiert. Für die Analyse steht ein Sample von 2563 Fällen (t_0_ = 2042 und t_1_ = 521) zur Verfügung. In Tab. 1 sind die Stichprobenverteilungen der zwei Surveys und der Population (60+) in NÖ dargestellt. Wichtig für die Analyse sind die Verteilung der Merkmale zwischen den beiden Surveys: Geschlecht, Altersgruppen und Haushaltsgröße sind ähnlich verteilt, etwas größer sind die Abweichungen bei den Bildungsabschlüssen. In Summe weisen die Strukturen aber eine akzeptable Passung auf.

**Table 1:** 

		t_0_		t_1_		Population^a^
		*n*	%	*n*	%	%
*Geschlecht*	Männlich	922	45	221	42	45
	Weiblich	1120	55	300	58	56
*Altersgruppen*	60–69	845	41	204	39	44
	70–79	766	38	191	37	35
	80–89	374	18	104	20	17
	90+	57	3	22	4	4
*Bildungsabschlüsse*	Max. Pflichtschule	282	14	90	17	*32*
	Lehre/mittlere Schule	1117	55	328	63	53
	Matura	423	21	64	12	8
	Hochschule	219	11	39	7	7
*Haushaltsgröße*	1	656	32	192	37	*45*
	2 oder mehr	1381	68	328	63	55

^a^Daten von 2018***–***2019 aus dem STATcube der Statistik Austria

Zur statistischen Prüfung (IBM SPSS 26) des Einsamkeitsniveaus zwischen den zwei Messzeitpunkten und den sozioökonomischen Gruppen wurden aufgrund der divergierenden Gruppengrößen und fehlender Varianzhomogenität acht Welch-ANOVAs mit Games-Howell Post-Hoc-Tests durchgeführt. Dafür wurden die acht Konstellationen – bestehend aus dem Zeitpunkt der Messung und je einem von acht sozidemografischen Faktoren – in insgesamt acht Gruppenvariablen (etwa Gruppenvariable 1: Frauen vor; Frauen während; Männer vor; Männer während der Pandemie) transformiert. Samplegröße und die große Anzahl an Kombinationen verhindern eine mehrfaktorielle Analyse mit allen Faktoren; entsprechend orientiert sich die Arbeit am Vorgehen wie bei Pieh et al. (2020) oder Parlapani et al. (2020).

Die Messung der Einsamkeit als abhängige Variable erfolgte via Kurzversion der de Jong Gierveld Einsamkeitsskala (de Jong Gierveld und van Tilburg 2006). Den RespondentInnen werden hierbei sechs Aussagen vorgelesen, die sie von „sehr zutreffend“ (1) bis „nicht zutreffend“ (4) einstufen sollen. Der additive Index erreicht Werte von 0 bis 18, wobei höhere Werte ein höheres Maß an Einsamkeit anzeigen.

Die unabhängigen Variablen sind aus statistischen Gründen in möglichst wenige Kategorien zusammengefasst worden (Tab. 2). Entsprechend einer gesonderten Analyse der Mittelwerte für Einsamkeit erfolgte die Bündelung des Familienstands in „ledig/geschieden/verwitwet“ (keine signifikanten Mittelwertsunterschiede) und „verheiratet“; die Haushaltsform wurde in „Ein-“ und „Mehrpersonenhaushalte“ zusammengefasst. Die Erfassung des formal höchsten Bildungsabschlusses orientiert sich an der, von der Statistik Austria gebräuchlichen und etwa im SILC (Statistik Austria 2019) angewandten, Nomenklatur „max. Pflichtschule“, „Lehre/mittlere Schule“ sowie „Matura/Universität“; aufgrund geringer Fallzahlen bei universitären Abschlüssen (siehe Tab. 1) wurde diese mit der Kategorie Matura zusammengefasst (die Korrelationsanalyse zeigt einen nahezu unveränderten Spearman-Rho mit Einsamkeit vor r_s0_ = −0,125 und nach Bündelung der Kategorien r_s1_ = −0,123). Der Gesundheitszustand wurde entlang des subjektiven Gesundheitsempfindens auf einer 5‑stufigen Skala abgefragt und in „sehr gut/gut“, „mittelmäßig“ und „schlecht/sehr schlecht“ eingeteilt (Korrelationsanalyse: r_s0_ = 0,322 zu r_s1_ = −0,333). Um die stark unterschiedlichen Gruppengrößen der drei folgenden Variablen auszugleichen, erfolgte jeweils eine Einteilung der Befragten in drei möglichst^1^ gleich große Gruppen (Terzile), welche je als „niedrig/klein“, „mittelmäßig/mittelgroß“ und „hoch/groß“ gelesen werden können. Um gleich große Terzilwerte der prä- und peri-pandemischen Stichprobe zu gewährleisten, erfolgte die Bildung über das gesamte Sample. In allen drei Fällen – Einkommen (r_s0_ = −0,248 zu r_s1_ = −0,241), Netzwerkgröße (r_s0_ = −0,418 zu r_s1_ = −0,392) und soziale Unterstützung (r_s0_ = −0,686 zu r_s1_ = −0,638) – bilden die Gruppierungen den Zusammenhang mit Einsamkeit weiterhin adäquat ab.

**Table 2:** 

UV	Indikator im Fragebogen	Kategorisierung für Analyse
*Geschlecht*	Frau; Mann	(1) „Frau“ und (2) „Mann“
*Familienstand*	Ledig, geschieden, verwitwet, verheiratet	(1) „Ledig/geschieden/verwitwet“ und (2) „verheiratet“
*Haushaltsform*	Anzahl der im Haushalt lebenden Personen	(1) „Ein-“ und (2) „Mehrpersonenhaushalt“
*Bildung*	Höchster formaler Bildungsabschluss	(1) „max. Pflichtschule“, (2) „Lehre/mittlere Schule“ sowie (3) „Matura/Universität“
*Gesundheitszustand*	Subjektive Gesundheit entlang 5‑teiliger Skala	(1) „sehr gut/gut“, (2) „mittelmäßig“ und (3) „schlecht/sehr schlecht“
*Einkommen*	Offene Frage nach Haushaltseinkommen, bei Verweigerung Zweitversuch über vorgegebene Kategorien	(1) „niedrig“, (2) „mittel“ und (3) „hoch“
*Netzwerkgröße*	Geschätzte Anzahl persönlich nahestehender Personen	(1) „klein“, (2) „mittel“ und (3) „groß“
*Soziale Unterstützung*	Additiver Index basierend auf Skala F‑SozU‑6 (Kliem et al. 2015)	(1) „niedrig“, (2) „mittel“, (3) „hoch“

## Ergebnisse

**Figure 1:**
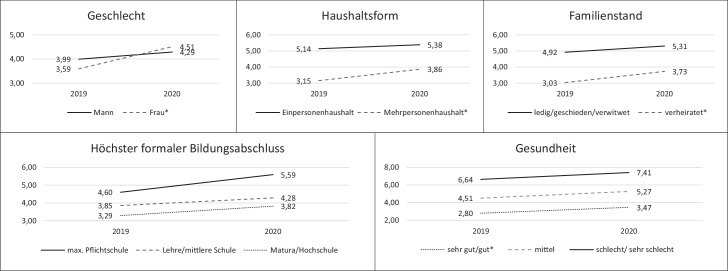

Sowohl vor als auch während der Pandemie ist ein geringes Einsamkeitsniveau bei den älteren Befragten zu konstatieren, wobei während der ersten Monate der Pandemie eine signifikante, wenn auch inhaltlich als gering einzustufende Zunahme stattgefunden hat: t_0_ (*M* = 3,77, *SD* = 3,37, *n* = 1845) gegenüber t_1_ (*M* = 4,42, *SD* = 3,58, *n* = 503); *t*(761,838) = −3,618, *p* = 0,000. Dies äußert sich auch darin, dass der Median von t_0_ = 3 auf t_1_ = 4 stieg. In Abb. 1 ist nun die nach soziodemografischen Faktoren nuancierte Entwicklung dargestellt, wobei zwei Aspekte visualisiert sind: Zum einen sind die Mittelwerte im Einsamkeitsniveau je Gruppe gelistet und es zeigen sich (teils signifikante) Unterschiede sowohl vor als auch während der Pandemie zwischen den Gruppen. Abseits von Personen mit großen sozialen Netzwerken ist im Vergleich der beiden Zeitpunkte innerhalb der Gruppen zumindest von einem leichten Anstieg der Einsamkeitsniveaus zu sprechen. Um zu ermitteln, ob es sich um signifikante Unterschiede handelt, wurden Post-Hoc-Tests durchgeführt. In Abb. 1 werden daher zum Zweiten signifikante Unterschiede durch ein *‑Zeichen (in der Legende) ausgewiesen, um anzuzeigen, dass es innerhalb der Merkmalsausprägung (etwa Frauen) zwischen den Zeitpunkten einen signifikanten Unterschied gibt. Solch einer lässt sich für Frauen – t_0_ (*M* = 3,59) zu t_1_ (*M* = 4,51) bzw. (*Mittelwertdifferenz MD*: 0,92; *p* = 0,000) –, verheiratete Personen (*MD*: 0,70; *p* = 0,01), Personen aus Mehrpersonenhaushalten (*MD*: 0,71; *p* = 0,000), ältere Befragte mit einem sehr guten/guten Gesundheitszustand (*MD*: 0,68; *p* = 0,000), bei Älteren mit mittelgroßen sozialen Netzwerken (*MD*: 0,75; *p* = 0,04) und bei mittlerer (*MD*: 1,09; *p* = 0,000) bzw. großer (*MD*: 0,58; *p* = 0,000) sozialer Unterstützung feststellen. Signifikant höhere Einsamkeitswerte sind entsprechend für jene Gruppen zu konstatieren (außer bei Personen mit umfangreichen Netzwerken und den Faktoren Bildung und Einkommen), welche vor der Pandemie die niedrigsten Einsamkeitswerte hatten (siehe die strichlierten Linien in Abb. 1). Auch wenn es sich um keine Paneldaten handelt, so lässt sich dies gut durch eine Line zwischen den zwei Zeitpunkten visualisieren – die strichlierten Linien weisen in Abb. 1 auf einen stärkeren (daher steileren) Anstieg hin.

Der steilere Anstieg bei Gruppen mit niedrigeren Einsamkeitswerten lässt eine Konvergenz innerhalb der sozioökonomischen Faktoren vermuten. In Tab. 3 sind daher die Post-Hoc-Tests je Messzeitpunkt zwischen den einzelnen Merkmalsausprägungen angegeben (*Mittelwertdifferenz MD* *=* J–I zum Zeitpunkt t_0_ oder t_1_). Hiermit soll geprüft werden, ob signifikante Unterschiede (etwa zwischen den Ausprägungen des Familienstands) vor der Pandemie auch während der Pandemie bestehen blieben. Allgemein zeigen sich teils deutliche Unterschiede zwischen den Ausprägungen: So hatten Personen mit einer hohen sozialen Unterstützung einen sehr niedrigen Einsamkeitswert vor der Pandemie im Vergleich zu jenen mit geringer sozialer Unterstützung (*M* *=* *1,54 versus M* *=* *6,43*), was sich in einer entsprechend hohen Mittelwertdifferenz ausdrückt (siehe Tab. 3: *MD* *=* *4,89, p* = 0,000). Im Messzeitpunkt während der Pandemie (t_1_) beträgt die Mittelwertdifferenz 4,66; der Unterschied ist ebenso signifikant und etwas geringer als zum Zeitpunkt t_0_. Dies deutet auf eine sehr geringe Annäherung der Einsamkeitswerte zwischen den Gruppen in der Pandemie hin. Das gleiche Muster findet sich bei den Faktoren Familienstand, Haushaltsform bzw. im Vergleich der niedrigsten und höchsten Ausprägung beim Haushaltseinkommen. Zu einer sehr geringen Ausweitung der Differenz bei niedrigster und höchster Ausprägung kam es hingegen bei Bildungsstand, Gesundheitszustand und Netzwerkgröße. Trotz relativ starker Zunahme der Einsamkeitswerte bei Frauen unterscheiden sich die Geschlechter zu beiden Messzeitpunkten nicht signifikant voneinander.

**Table 3:** 

		*MD* im Vergleich zu … (J–I)			*MD* im Vergleich zu … (J–I)	
–	–	(J) *Mann*		–	(J) *verheiratet*	
t0	(I) *Frau*	0,4 *(p* *=* *0,057)*		(I) *ledig/geschieden/verwitwet*	**1,89** *(p* *=* *0,000)*	
t1		−0,22 *(p* *=* *0,899)*			**1,58** *(p* *=* *0,000)*	
–	–	(J) Mehrpersonenhaushalt		–	(J) *Lehre/mittlere Schule*	(J) *Matura/Hochschule*
t0	(I) Einpersonenhaushalt	**1,99** *(p* *=* *0,000)*		(I) *max. Pflichtschule*	**0,75*** (p* *=* *0,039)*	**1,31** *(p* *=* *0,000)*
t1		**1,52** *(p* *=* *0,000)*			1,31 *(p* *=* *0,083)*	**1,77*** (p* *=* *0,014)*
–	–	(J) *mittleres*	(J) *hohes*	–	(J) mittel	(J) gut/sehr gut
t0	(I) *niedriges* Haushaltseinkommen	**1,40** *(p* *=* *0,000)*	**2,01** *(p* *=* *0,000)*	(I) schlechte/sehr schlechte Gesundheit	**2,13** *(p* *=* *0,000)*	**3,84** *(p* *=* *0,000)*
t1		**1,55** *(p* *=* *0,001)*	**1,88** *(p* *=* *0,001)*		2,14 *(p* *=* *0,051)*	**3,94** *(p* *=* *0,000)*
–	–	(J) *mittlere*	(J) *große*	–	(J) *mittlere*	(J) *hohe*
t0	(I) *geringe* Netzwerkgröße	**2,24** *(p* *=* *0,000)*	**3,32** *(p* *=* *0,000)*	(I) *geringe* soziale Unterstützung	**2,97** *(p* *=* *0,000)*	**4,89** *(p* *=* *0,000)*
t1		**1,67** *(p* *=* *0,000)*	**3,73** *(p* *=* *0,000)*		**2,18** *(p* *=* *0,000)*	**4,66** *(p* *=* *0,000)*

Lesehilfe: Differenz der Geschlechter in t_0_ = Mann (J) *M:3,99* minus Frau (I) *M:3,59* = *MD von 0,4 für t*_*0*_

Lesehilfe: Im Vergleich der Mittelwertdifferenz beispielsweise der beiden Haushaltsformen zum Zeitpunkt t_0_ (*MD:1,99*) und t_1_ (*MD:1,52*) lässt sich aufgrund der kleineren Differenz in t_1_ von einer geringfügigen Konvergenz sprechen

Fett gedruckte Werte unterscheiden sich signifikant (Games-Howell Post-Hoc-Test) voneinander; *p* < 0,05

## Diskussion

Die Analysen zeigen zum Ersten das bekannte Bild von Unterschieden im Einsamkeitsniveau entlang der sozioökonomischen Faktoren und schließen damit an vorangegangene Arbeiten an (Cohen-Mansfield et al. 2016; Luhmann und Hawkley 2016; Victor et al. 2005). Zum Zweiten ist zumindest von einem Gleichbleiben, eher aber von einer leichten Zunahme der Einsamkeit während der ersten Monate der Pandemie in den meisten geprüften Gruppen auszugehen. Auch wenn die Ergebnisse nicht immer statistisch signifikant sind, deutet der, in der Abbildung weitestgehend steigende Trend auf einen möglichen Fahrstuhleffekt hin. Die vorliegende Trendanalyse bekräftigt damit das internationale Phänomen einer (wenn auch nur geringen) Zunahme der Einsamkeit bei älteren Menschen während der Pandemie (Entringer et al. 2020; Heidinger und Richter 2020; Luchetti et al. 2020; van Tilburg et al. 2020). Aufgrund des allgemeinen Niveauanstiegs blieben (signifikante) Differenzen innerhalb der sozioökonomischen Faktoren auch während der Pandemie bestehen – hiermit schließt die Arbeit an Wong et al. (2020), Stolz et al. (2020), Whatley et al. (2020) sowie Stickley et al. (2021) an, welche signifikante Zusammenhänge zwischen Einsamkeit und Haushaltsform, Gesundheit sowie dem Einkommen auch während der Pandemie konstatieren. Der, in den genannten Arbeiten nachgewiesene, signifikante Einfluss des Geschlechts lässt sich durch die vorliegende Arbeit nicht bestätigen, was auf das fehlende multivariate Design zurückzuführen sein könnte. Der verhältnismäßig starke und signifikante Niveauanstieg bei Frauen während der Pandemie dürfte das Resultat des bereits bei Wickens et al. (2021) berichteten, überproportional bei älteren Frauen gehäuft auftretenden Einsamkeitsgefühls sein. Bemerkenswert sind zudem die Stabilität bzw. der potenzielle Rückgang der Einsamkeit (Ergebnis jedoch nicht signifikant) bei älteren Menschen mit besonders großen sozialen Netzwerken. Einerseits kommt der Größe sozialer Netzwerke eine protektive Funktion gegenüber Einsamkeit zu (Cohen-Mansfield et al. 2016); zum anderen ist denkbar, dass die Ungewissheit in den ersten Monaten der Pandemie Nachfragen der Unversehrtheit der eigenen Kontakte initiierte und so zu einem – wenn auch nur über die Telekommunikation hergestellten – gesteigerten sozialen Kontakt beigetragen hatte; dafür spricht, dass nach Angaben der Befragten die Frequenz des telefonischen Kontakts gegenüber der Zeit vor der Pandemie anstieg (Kolland et al. 2020). Bedenkt man, dass es sich bei Einsamkeit um die subjektiv empfundene Diskrepanz zwischen der erwarteten und tatsächlichen Qualität und Quantität handelt, so lässt sich hier von einer positiven Bilanzierung sprechen.

Zusammenfassend ist die Entwicklung der Einsamkeit wohl nicht völlig homogen verlaufen, so lässt sich nur bei vor der Pandemie relativ gut situierten Gruppen ein signifikanter Einsamkeitsanstieg konstatieren. Jedoch sind als drittes Ergebnis die aufgedeckten Mittelwertdifferenzen für das Sample 2019 und 2020 sehr ähnlich – die Unterschiede zwischen den Zeitpunkten bleiben inhaltlich betrachtet also sehr gering. Vermutlich haben sich mehrere Effekte überlagert: Betrachtet man etwa die Ausgaben für Cafés und Restaurants (Statistik Austria 2017), so wäre davon auszugehen, dass Haushalte mit höherem Einkommen durch den Lockdown stärker in ihrem Lebensstil und einem Verlust von Orten des sozialen Kontakts betroffen waren und sich dies in einer proportional größer empfundenen Diskrepanz der gewohnt erwarteten und tatsächlichen sozialen Kontakte äußern hätte müssen. Umgekehrt dürften aber auch gegenläufige Mechanismen wirken: So besitzen ältere Menschen mit höherem Einkommen eine größere Technikbereitschaft (Beil et al. 2015) und haben so eine Möglichkeit, den Kontaktverlust zu kompensieren – dies könnte den Einsamkeitstrend abgeschwächt haben. Unterschiedliche Entwicklungen des Einsamkeitsempfindens wären argumentativ also durchaus naheliegend und es bedarf empirischer Studien, um die Mechanismen näher zu bestimmen. Für die vorliegende Studie lässt sich zusammenfassen, dass sich die bereits vor der Pandemie bestehenden Unterschiede weitgehend auf einem etwas höheren Niveau erhalten haben.

## Footnotes

1 Trotzdem auftretende Unterschiede der Gruppengröße sind in einer geringen Zahl an faktisch vorhandenen Ausprägungen der Variablen und dem Umstand, dass gleichgroße Werte nicht auf verschiedene Ntile-Gruppen aufgeteilt werden, begründet.
